# A protein domain-oriented approach to expand the opportunities of therapeutic exon skipping for *USH2A*-associated retinitis pigmentosa

**DOI:** 10.1016/j.omtn.2023.05.020

**Published:** 2023-05-20

**Authors:** Renske T.W. Schellens, Sanne Broekman, Theo Peters, Pam Graave, Lucija Malinar, Hanka Venselaar, Hannie Kremer, Erik De Vrieze, Erwin Van Wijk

**Affiliations:** 1 Department of Otorhinolaryngology, Hearing and Genes, Radboud University Medical Center, 6525GA Nijmegen, the Netherlands; 2 Donders Institute for Brain, Cognition and Behaviour, 6500 GL Nijmegen, the Netherlands; 3 Department of Human Genetics, Radboud University Medical Center, 6525 GA Nijmegen, the Netherlands; 4 Center for Molecular and Biomolecular Informatics, Radboud University Medical Center, 6525 GA Nijmegen, the Netherlands

**Keywords:** MT: Oligonucleotides: Therapies and Applications, *USH2A*, antisense oligonucleotides, CRISPR-Cas9, dual exon skipping, photoreceptors, retinitis pigmentosa, zebrafish

## Abstract

Loss-of-function mutations in *USH2A* are among the most common causes of syndromic and non-syndromic retinitis pigmentosa (RP). We previously presented skipping of *USH2A* exon 13 as a promising treatment paradigm for *USH2A*-associated RP. However, RP-associated mutations are often private, and evenly distributed along the *USH2A* gene. In order to broaden the group of patients that could benefit from therapeutic exon skipping strategies, we expanded our approach to other *USH2A* exons in which unique loss-of-function mutations have been reported by implementing a protein domain-oriented dual exon skipping strategy. We first generated zebrafish mutants carrying a genomic deletion of the orthologous exons of the frequently mutated human *USH2A* exons 30–31 or 39–40 using CRISPR-Cas9. Excision of these in-frame combinations of exons restored usherin expression in the zebrafish retina and rescued the photopigment mislocalization typically observed in *ush2a* mutants. To translate these findings into a future treatment in humans, we employed *in vitro* assays to identify and validate antisense oligonucleotides (ASOs) with a high potency for sequence-specific dual exon skipping. Together, the *in vitro* and *in vivo* data demonstrate protein domain-oriented ASO-induced dual exon skipping to be a highly promising treatment option for RP caused by mutations in *USH2A*.

## Graphical abstract

**Figure:**
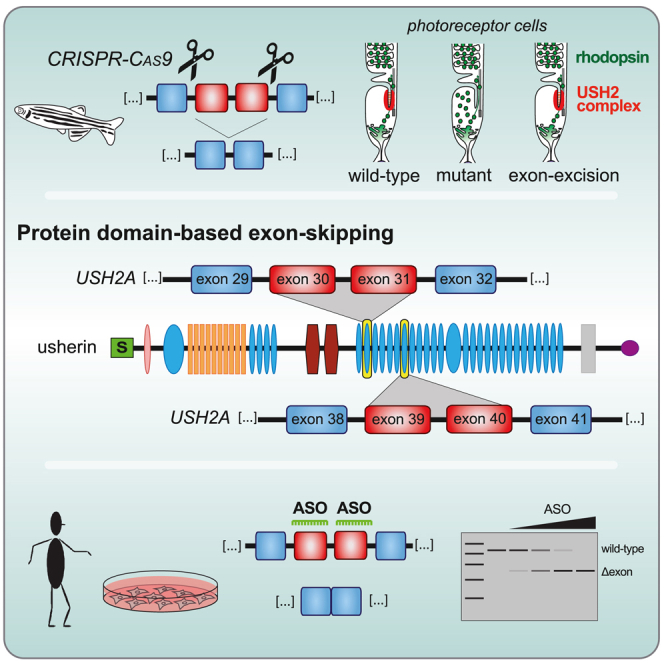

## Teaser

Van Wijk and colleagues showed that a protein domain-based skipping of (multiple) exons, based on a powerful combination of 3D bioinformatic modeling with *in vitro* and *in vivo* models, is a highly promising treatment paradigm for retinitis pigmentosa caused by pathogenic variants in the *USH2A* gene.

## Introduction

Retinitis pigmentosa (RP) is a genetically and clinically heterogeneous condition that is currently still largely untreatable. Patients usually present with a progressive loss of visual function that initially manifests with night blindness and visual field constriction during adolescence. This can progress toward the loss of central vision and ultimately legal blindness in later stages of life.^1^ With a predicted overall prevalence of 1 in 4,000 individuals, RP is estimated to affect almost 2 million individuals worldwide.^2^ Mutations in *USH2A* are the most frequent cause of RP with an autosomal recessive mode of inheritance (arRP), accounting for up to 23% of all arRP cases.^3^ Besides leading to non-syndromic RP, mutations in *USH2A* can also result in Usher syndrome. Patients suffering from Usher syndrome experience a double sensory impairment, a combination of RP and congenital hearing impairment, making the development of a therapy to halt or delay their progressive vision loss even more urgent. The delayed onset and slowly progressive nature of *USH2A*-associated RP, and the often early genetic diagnosis of Usher syndrome through genetic testing after the observation of congenital hearing impairment, provides ample time for therapeutic intervention.

The *USH2A* gene, located on chromosome 1q41, spans approximately 800 kb and encodes two different isoforms of usherin. The large usherin isoform (isoform B) consists of 5,202 amino acids and is encoded by 72 exons. This isoform is predominantly expressed in photoreceptor cells of the retina and hair cells of the cochlea.^4^^,^^5^^,^^6^^,^^7^^,^^8^^,^^9^^,^^10^ The short isoform (isoform A) consists of 1,551 amino acids encoded by a transcript built up by the initial 21 5′ exons, and is expressed more widely.^4^^,^^11^^,^^12^ In total, over 600 different mutations have been identified in the transcript encoding the large isoform of usherin. As these mutations are mostly private and distributed all over the gene, the development of a mutation-independent therapy is preferred to eventually treat a significant group of patients (*USH2A* LOVD mutation database, https://databases.lovd.nl/shared/variants/USH2A/unique).^13^

The size of the usherin-encoding sequence (15.6 kb) severely hampers the development of conventional gene augmentation therapy, as the protein-encoding sequence by far exceeds the packaging capacity of the currently preferred vehicles for retinal gene delivery.^14^ An attractive alternative approach is antisense oligonucleotide (ASO)-induced splice modulation. In this approach, ASOs are applied to correct aberrant pre-mRNA splicing or to remove native in-frame exons that harbor recurrent loss-of-function mutations. Both approaches aim to restore the original open reading frame and protein function. By targeting the pre-mRNA, ASOs are able to transiently modulate transcripts without altering transcript levels. We previously presented ASO-induced splice correction as a promising treatment option for the correction of aberrant mRNA splicing caused by the deep intronic c.7595-2144A>G mutation in *USH2A*.^15^ Additionally, ASOs are particularly suited to induce the skipping of native in-frame exons that contain disease-associated mutations. In-frame exon skipping is particularly interesting for large genes encoding (structural) proteins that contain a series of repetitive protein domains, such as *USH2A, DMD,* and *NOTCH3*.

More recently, we published the first ASO-based exon skipping therapy for mutations affecting *USH2A* exon 13.^16^ This single exon skipping therapy reached the clinical phase and resulted in a concordant benefit in multiple parameters of visual function (i.e., visual acuity, static perimetry, and retinal imaging) without induction of any serious adverse events (Trial # NCT03780257).^17^ Skipping of exon 13 was not intended to result in the removal of a single protein domain. Instead, it resulted in the loss of 4 EGF-lam domains and the formation of one EGF-like hybrid domain. The resulting shortened protein was shown to retain function. The amount of single *USH2A* exons that encode (a) full protein domain(s) and of which skipping is predicted to result in restoration of the open reading frame, is minimal. An alternative strategy is to skip a combination of exons, consisting of a total number of nucleotides that is divisible by three, that together encode a complete protein domain. Such a multiple exon skipping strategy increases the therapeutic options for *USH2A*-associated RP dramatically, and has already been proven to be successful in patient-derived cell models for Duchenne muscular dystrophy (DMD) and CADASIL.^18^^,^^19^

In this study, we explored dual exon skipping as a future treatment option for patients with RP caused by mutations in *USH2A*. Based on *in silico* protein domain analysis, and the reported presence of multiple RP-associated protein-truncating mutations, we opted to target exons 30–31 and exons 39–40 of human *USH2A*. Calculations based on available carrier frequencies in the general population for the various reported pathogenic variants in exons 30–31 and exons 39–40 (gnomAD v2.1.1) indicate that worldwide ∼22,000 individuals and ∼9,500 individuals suffer from *USH2A*-associated disease caused by a mutation in these exons, respectively.

Skipping of *USH2A* exons 30–31 or *USH2A* exons 39–40 was predicted to result in a transcript encoding a shortened protein that lacks exactly one of the repetitive fibronectin type III (FN3) domains. To model the functional consequences of our exon skipping approach, we employed CRISPR-Cas9 and excised the orthologous target exons from the zebrafish genome. This resulted in the *ush2a*^Δexon30-31^ and *ush2a*^Δexon39-40^ zebrafish lines in which usherin expression was restored in photoreceptors, and both the localization of rhodopsin to the photoreceptor outer segment and the formation of the USH2 protein complex at the periciliary membrane were rescued. To translate these findings into a future treatment in humans, we designed and *in vitro-*validated ASOs with a high potency for sequence-specific dual exon skipping. Together, our data suggest that a carefully designed dual exon skipping approach, in which two exons remove a single protein domain, could be highly promising as a future treatment option for *USH2A*-associated RP.

## Results

### *USH2A* exons 30–31 and *USH2A* exons 39–40 are promising targets for exon skipping

**Figure 1:**
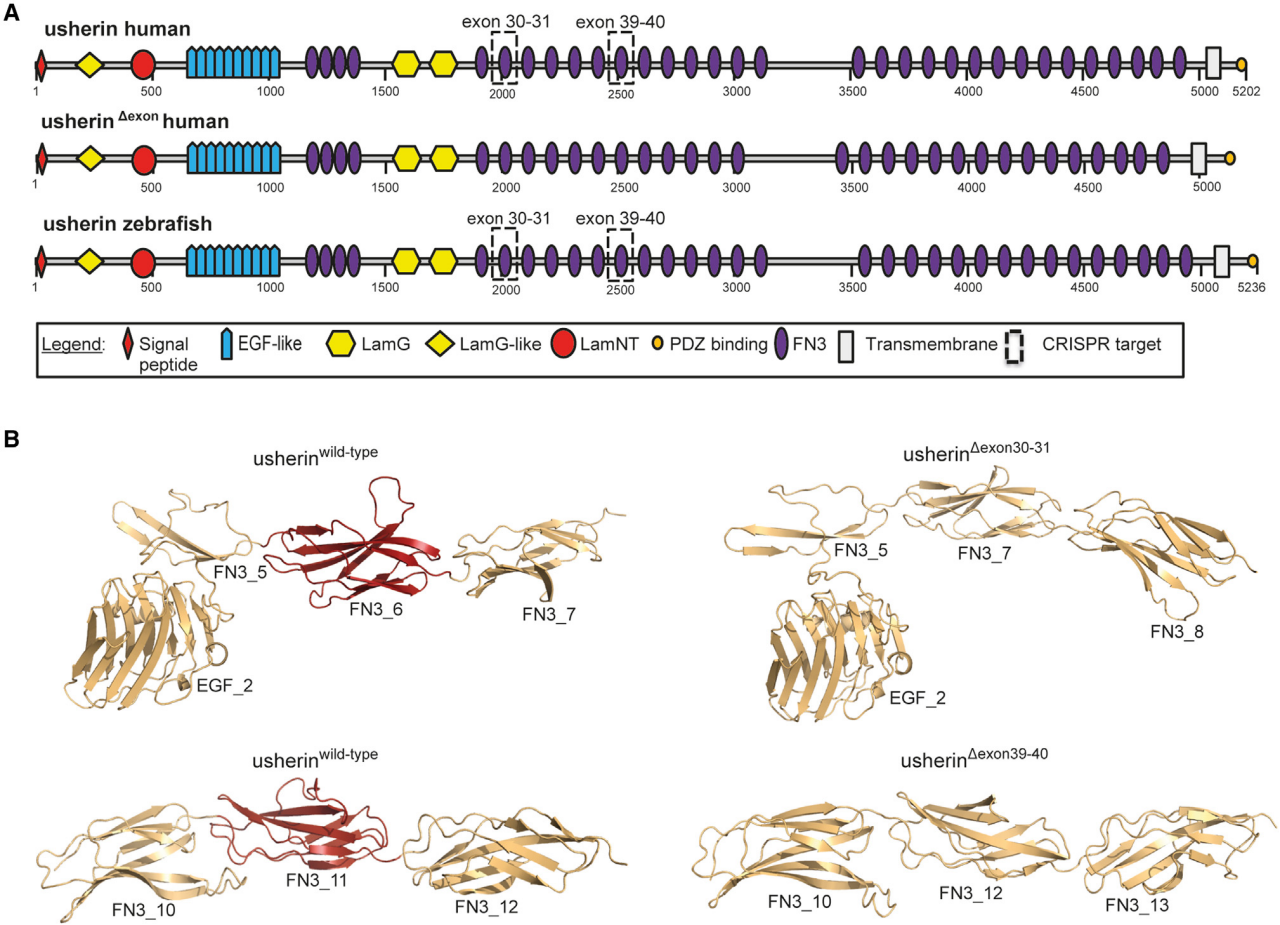
*In silico* modeling of usherin domain architecture after *USH2A* exon 30–31 and exon 39–40 skipping (A) Schematic representation of the domain architecture of the large isoform (isoform B) of human and zebrafish usherin. Isoform B of human and zebrafish usherin are composed of the same repetitive domain architecture that includes a signal peptide, a laminin G-like domain (LamG-like), a laminin N-terminal domain (LamNT), 10 EGF-like motifs, four fibronectin type III (FN3) domains, two laminin G domains (LamG), 28 additional FN3 domains, one transmembrane domain, and a short intracellular region with a C-terminal class I PDZ binding motif. Skipping of exons 30–31 and exons 39–40 both result in the absence of one FN3 domain. The domains that are lost in human and zebrafish usherin are indicated with dashed boxes. Both proteins that result from exon skipping are composed of the same protein domain architecture (visualized as usherin^Δexon^). Numbers indicate amino acids. (B) For both skipping of *USH2A* exons 30–31 and exons 39–40, 3D homology modeling predicts the removal of exactly one FN3 domain without disturbing the folding of neighboring FN3 domains. The part of usherin that is encoded by the targeted exons is depicted in red.

*USH2A* exons 30 and 31 (306 nucleotides) and *USH2A* exons 39 and 40 (294 nucleotides) both encode exactly one FN3 domain. In addition, many unique loss-of-function mutations have been reported in those exons, making them important targets for exon skipping (*USH2A* LOVD mutation database, https://databases.lovd.nl/shared/variants/USH2A/unique). Skipping these two combinations of exons will maintain the open reading frame of the *USH2A* transcript, and is predicted to result in the production of a slightly shortened protein lacking exactly one FN3 domain (Figure 1A). Three-dimensional homology modeling predicted human usherin^Δexon30-31^ and usherin^Δexon39-40^ to show a nearly identical protein domain structure in which folding of neighboring FN3 domains is not disturbed (Figure 1B).

### Generation of the *ush2a*^*Δexon30-31*^ and *ush2a*^*Δexon39-40*^ zebrafish line using CRISPR-Cas9 technology

We and others have previously established the translational value of zebrafish models to study the retinal phenotype of Usher syndrome.^6^^,^^16^^,^^20^ Human and zebrafish usherin share a similar domain architecture and an overall sequence identity of 52%.^6^ The protein regions encoded by zebrafish and human *USH2A* exons 30–31 and exons 39–40 show a 61% and 52% sequence identity, respectively. Similar to the human situation, the in-frame deletion of zebrafish *ush2a* exons 30–31 or exons 39–40 is predicted to result in a shortened protein (usherin^Δexon30-31^ or usherin^Δexon39-40^) from which exactly one FN3 domain is lost (Figure 1A).

**Figure 2:**
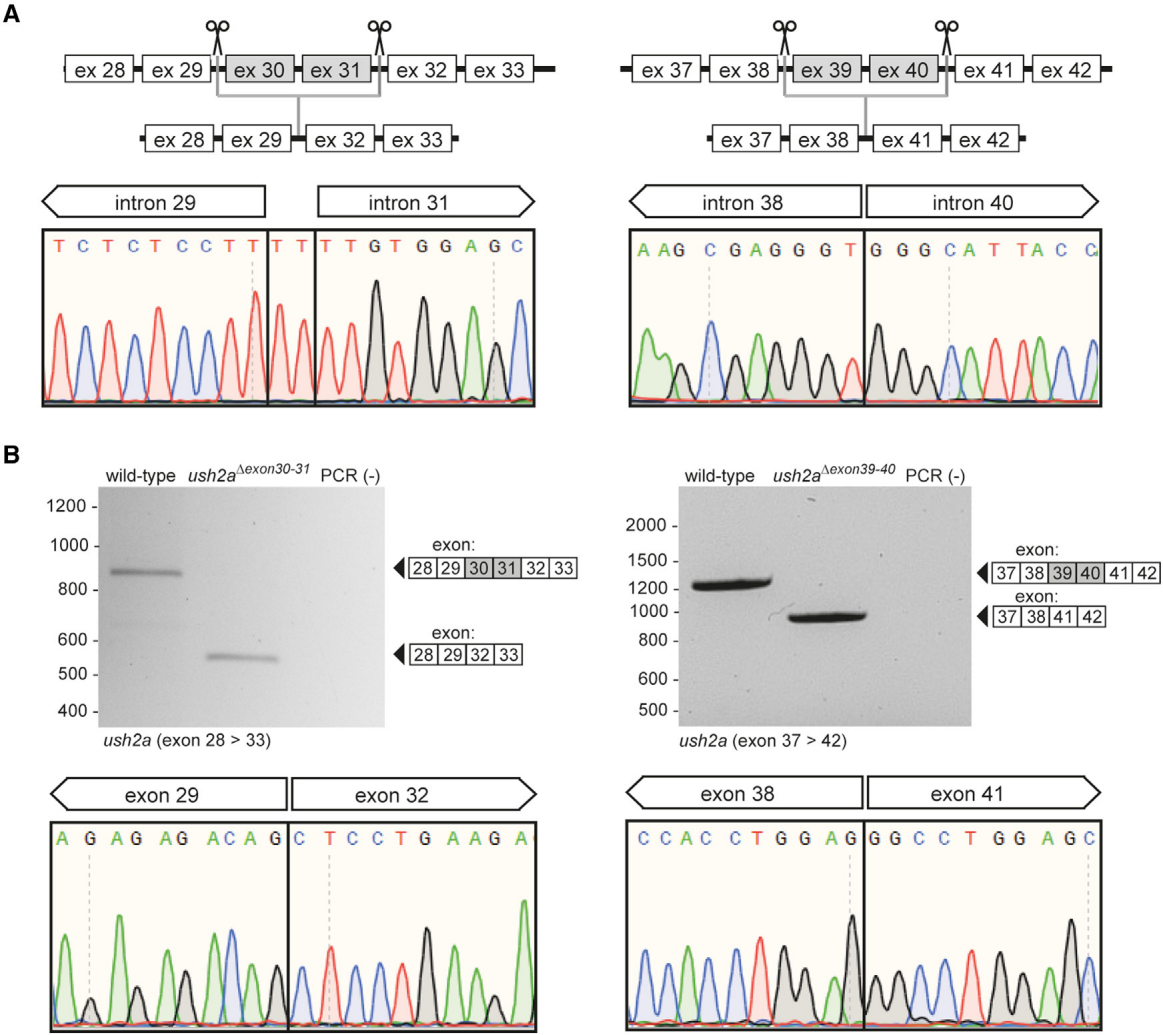
Design and characterization of the *ush2a*^*Δexon30-31*^ and *ush2a*^*Δexon39-40*^ zebrafish line (A) Schematic representation of the exon-excision approach. Sanger sequencing confirmed the presence of the anticipated excisions in injected embryos (1 day post fertilization [dpf]). Excision of the genomic region containing *ush2a* exons 30 and 31 resulted in the insertion of two nucleotides (TT) at the repair junction. (B) RT-PCR analysis revealed the absence of *ush2a* exons 30 and 31 in *ush2a*^*Δexon30-31*^ larvae and the absence of exons 39 and 40 in *ush2a*^*Δexon39-40*^ larvae (5 dpf). Sanger sequencing of the *ush2a*^*Δexon30-31*^ and *ush2a*^*Δexon39-40*^ amplicons confirmed the absence of the target exons from the transcript.

To assess the effects of dual exon skipping therapy on usherin function, we adopted CRISPR-Cas9 technology to generate stable zebrafish lines from which the genomic regions encompassing *ush2a* exons 30–31 or *ush2a* exons 39–40 were specifically excised. For this, Cas9 protein and two single guide RNAs (sgRNAs), one targeting the genomic region upstream and one targeting the genomic region downstream of the exonic targets, were injected in fertilized embryos (Figure 2A). Correct exon excision was confirmed by genomic PCR and Sanger sequencing. Stable homozygous zebrafish exons 30–31 and exons 39–40 excision lines were bred from germline-positive founder fish, and designated *ush2a*^*Δexon30-31*^ and *ush2a*^*Δexon39-40*^, respectively. Homozygous *ush2a*^*Δexon30-31*^ and *ush2a*^*Δexon39-40*^ fish were viable and did not display any abnormalities in overall body morphology, development, or swimming behavior.

To determine the effect of the excision of the target exons from the zebrafish genome at transcript level, total RNA was isolated from pairs of homozygous *ush2a*^*Δexon30-31*^ larvae and homozygous *ush2a*^*Δexon39-40*^ larvae. RT-PCR analysis using forward primers two exons upstream of the target exons, and reverse primers two exons downstream of the target exons detected shortened PCR fragments in both *ush2a*^*Δexon30-31*^ and *ush2a*^*Δexon39-40*^ zebrafish in the absence of any clear alternatively spliced *ush2a* transcripts (Figure 2B). Sanger sequencing confirmed the presence of the expected *ush2a* transcript exclusively lacking the anticipated target exons from the *ush2a* transcripts derived from the *ush2a*^*Δexon30-31*^ and *ush2a*^*Δexon39-40*^ larvae.

### Dual exon excision restores usherin expression in genetically modified zebrafish

**Figure 3:**
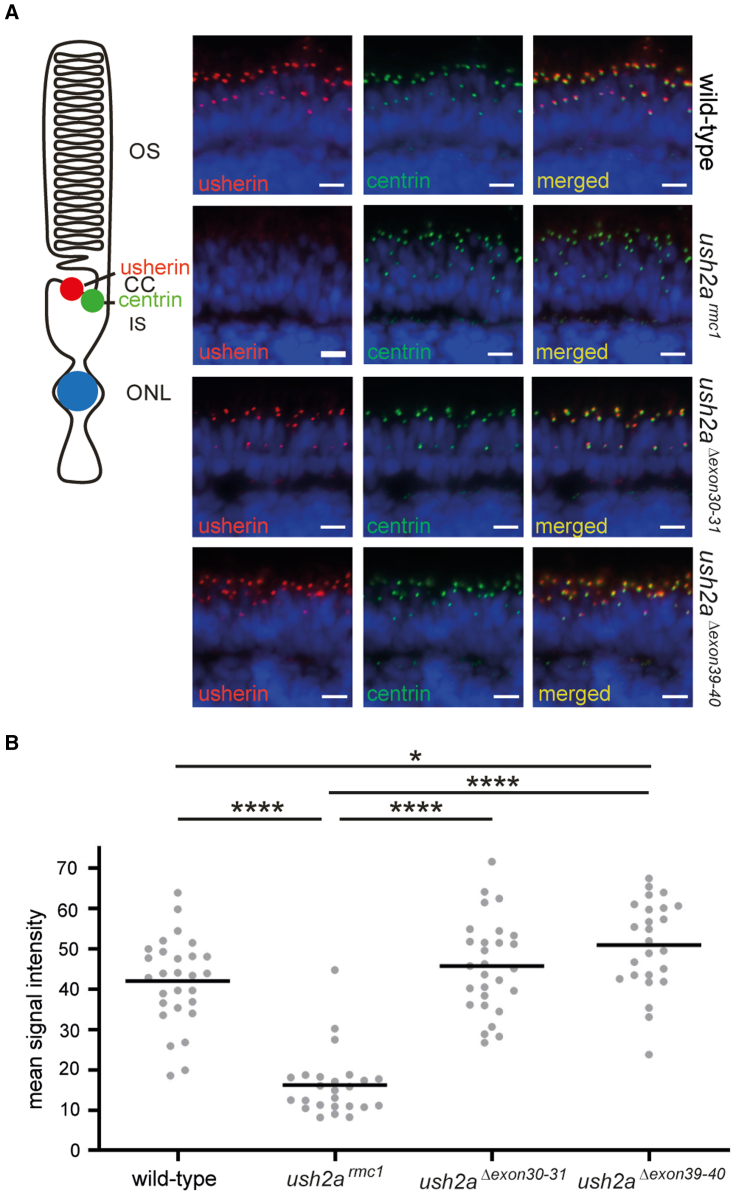
Visualization of usherin on retinal sections of wild-type, *ush2a*^*rmc1*^, *ush2a*^*Δexon30-31*^, and *ush2a*^*Δexon39-40*^ zebrafish (A) Retinal cryosections of wild-type, *ush2a*^*rmc1*^, *ush2a*^*Δexon30-31*^, and *ush2a*^*Δexon39-40*^ larvae (5 dpf) were stained with antibodies directed against usherin (red) and centrin (green). Nuclei are counterstained with DAPI (blue). No usherin signal was detected in the retina of *ush2a*^*rmc1*^ zebrafish, whereas in the retinas of wild-type, *ush2a*^*Δexon30-31*^, and *ush2a*^*Δexon39-40*^ larvae, usherin was present at the photoreceptor periciliary region, in close proximity to centrin. Scale bar, 10 μm. CC, connecting cilium; IS, inner segment; ONL, outer nuclear layer; OS, outer segment. (B) Quantification of anti-usherin signal intensity at the periciliary region. Individual data points represent the mean fluorescence intensity of anti-usherin staining at the periciliary region of all photoreceptors of a single, central section of one larval zebrafish eye. Horizonal bars depict the mean signal intensity within a genotype (n = 25–28 eyes). Data were analyzed using one-way ANOVA followed by Tukey’s multiple comparison test. ∗p ≤ 0.05. ∗∗∗∗p ≤ 0.0001.

To investigate whether the excision of the target exons resulted in the translation and correct localization of usherin^Δexon30-31^ and usherin^Δexon39-40^ in photoreceptor cells, an immunohistochemical analysis was performed. In the wild-type zebrafish retina, usherin is expressed at the periciliary region of photoreceptors.^6^^,^^15^^,^^16^ Antibodies directed against the intracellular region of usherin and antibodies directed against the connecting cilium marker centrin were used to co-stain unfixed retinal cryosections of 5 days post fertilization (dpf) wild-type, *ush2a*^*rmc1*^, *ush2a*^*Δexon30-31*^, and *ush2a*^*Δexon39-40*^ zebrafish larvae (Figure 3A). As expected, usherin was absent from photoreceptors of *ush2a*^*rmc1*^ larvae. Usherin^Δexon30-31^ and usherin^Δexon39-40^ localized at the photoreceptor periciliary region, adjacent to the connecting cilium marker centrin, similar to the localization of usherin in strain- and age-matched wild-type larvae. The mean intensity of the anti-usherin fluorescence signals at all periciliary regions in the middle section of each eye, was quantified using an automated Fiji script. This analysis demonstrates that excision of exons 30–31 or exons 39–40 leads to the generation of usherin at a level comparable to wild type with a correct subcellular localization (Figure 3B). More specifically, statistically significant differences were not found between anti-usherin fluorescent signal intensities in the wild-type (42.05 ± 10.90) and *ush2a*^*Δexon30*-31^ (45.77 ± 11.50) retina (p = 0.55), whereas the anti-usherin fluorescent signal intensity increased significantly in the *ush2a*^*Δexon39*-40^ retina (50.98 ± 11.16) as compared with the wild-type retina (42.05 ± 10.90). However, nearly all usherin intensities measured in *ush2a*^*Δexon39-40*^ larvae are within the range of the fluorescent signal intensity observed in the wild-type larvae.

### Dual exon excision does not alter Adgrv1 localization zebrafish photoreceptors

**Figure 4:**
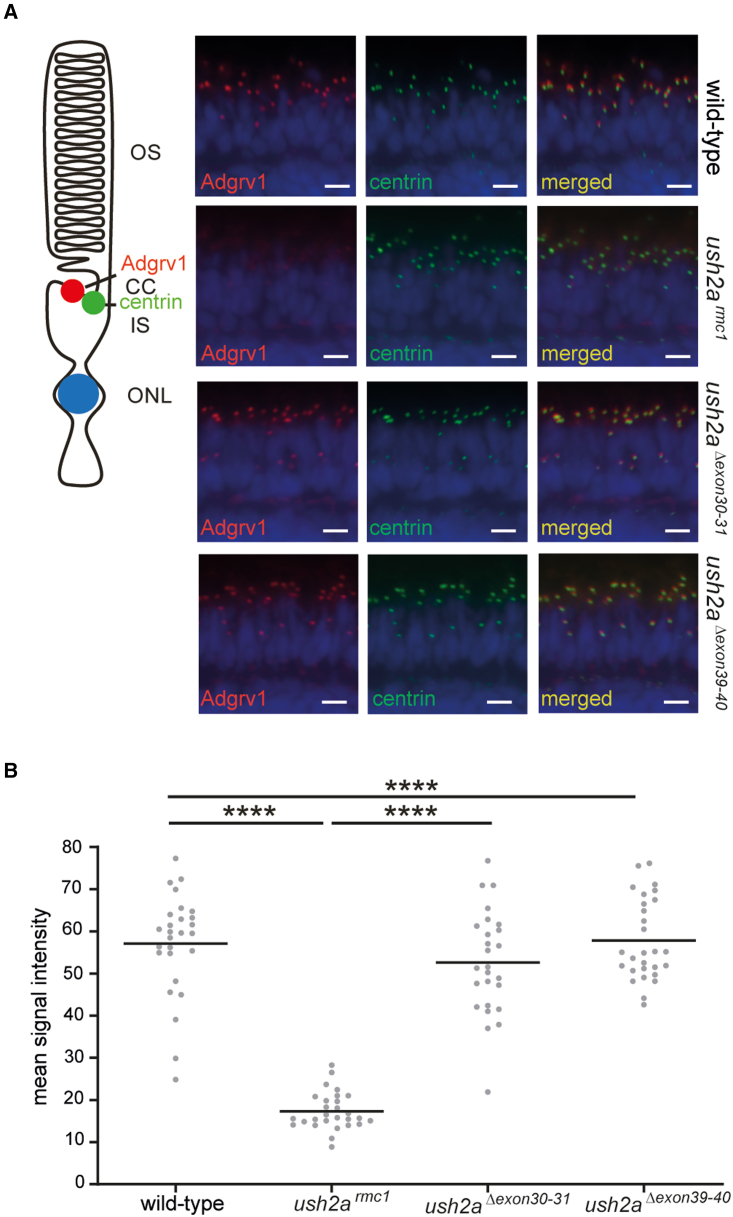
Visualization of Adgrv1 in retinal sections of wild-type, *ush2a*^*rmc1*^, *ush2a*^*Δexon30-31*^, and *ush2a*^*Δexon39-40*^ zebrafish (A) Retinal cryosections of wild-type, *ush2a*^*rmc1*^, *ush2a*^*Δexon30-31*^, and *ush2a*^*Δexon39-40*^ larvae (5 dpf) stained with antibodies directed against Adgrv1 (red) and centrin (green). Nuclei are counterstained with DAPI (blue). A reduced Adgrv1 signal was detected in *ush2a*^*rmc1*^ zebrafish retinas, whereas in the retinas of wild-type, *ush2a*^*Δexon30-31*^, and *ush2a*^*Δexon39-40*^ larvae, usherin was present adjacent to the centrin immunoreactivity. Scale bar, 10 μm. CC, connecting cilium; IS, inner segment; ONL, outer nuclear layer; OS: outer segment. (B) Quantification of anti-Adgrv1 signal intensity at the periciliary region. Individual data points represent the mean fluorescence intensity of anti-Adgrv1 staining at the periciliary region of all photoreceptors of a single, central section of one larval zebrafish eye. Horizonal bars depict the mean signal intensity within a genotype (n = 26–28 eyes). Data were analyzed using one-way ANOVA followed by Tukey’s multiple comparison test. ∗∗∗∗p ≤ 0.0001.

The correct localization of shortened usherin indicates that the FN3 domains encoded by exons 30–31 and exons 39–40 can be skipped without interfering with usherin expression and subcellular localization in zebrafish photoreceptors. As a next step, the effect of the excision of the target exons on the USH2 protein complex integrity was analyzed. In humans, usherin and the other known USH2 proteins, whirlin and ADGRV1, form a dynamic USH2 protein complex.^21^^,^^22^ We previously showed that defects of usherin lead to reduced levels of the USH2 complex members, whirlin and Adgrv1, at the photoreceptor periciliary membrane of *ush2a*^*rmc1*^ zebrafish larvae. To analyze whether the excision of the target exons resulted in the assembly of the USH2 protein complex in zebrafish photoreceptors, antibodies directed against the N-terminal region of Adgrv1 and antibodies directed against centrin were used to stain unfixed retinal cryosections of 5 dpf wild-type, *ush2a*^*rmc1*^, *ush2a*^*Δexon30-31*^, and *ush2a*^*Δexon39-40*^ larvae (Figure 4A). The mean intensity of the anti-Adgrv1 fluorescence signals at all periciliary regions in the middle section of each eye, was quantified using an automated Fiji script (Figure 4B).^6^ Anti-Adgrv1 immunoreactivity was indeed significantly reduced in photoreceptors of *ush2a*^*rmc1*^ larvae (17.33 ± 4.42) as compared with wild-type controls (57.14 ± 12.12) (p < 0.0001). In contrast, Adgrv1 expression at the photoreceptor periciliary region was restored in *ush2a*^*Δexon30-31*^ larvae (52.59 ± 12.33) and *ush2a*^*Δexon39-40*^ larvae (57.82 ± 9.67) as compared with *ush2a*^*rmc1*^ larvae (17.33 ± 4.42) (p < 0.0001 [*ush2a*^*Δexon30-31*^] and p < 0.0001 [*ush2a*^*Δexon39-40*^]). These data suggest that, similar to wild-type usherin, usherin^Δexon30-31^ and usherin^Δexon39-40^ are able to scaffold the USH2 protein network at the photoreceptor periciliary region.

### Dual exon excision restores rhodopsin trafficking in genetically modified zebrafish

**Figure 5:**
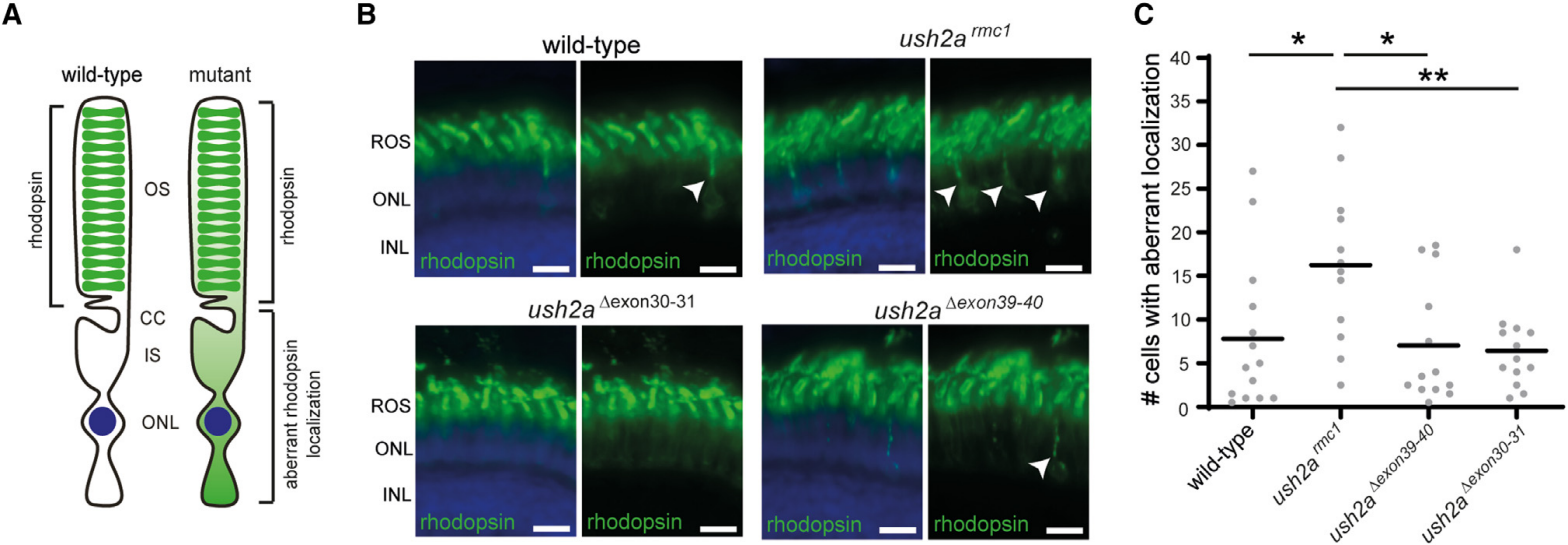
Visualization of rhodopsin in retinal sections of wild-type, *ush2a*^*rmc1*^, *ush2a*^*Δexon30-31*^, and *ush2a*^*Δexon39-40*^ zebrafish (A) In the photoreceptors of wild-type zebrafish, rhodopsin predominantly localizes to the outer segments, whereas in photoreceptors of mutant zebrafish, rhodopsin is defectively localized. (B) Retinal cryosections of larvae (6 dpf) were analyzed for rhodopsin (green) localization. Nuclei were counterstained with DAPI (blue). In the retinas of wild-type, *ush2a*^*Δexon30-31*^, and *ush2a*^*Δexon39-40*^ larvae, rhodopsin was predominantly present in the photoreceptor outer segments, whereas in *ush2a*^*rmc1*^ retinas rhodopsin signal was also detected in the photoreceptor cell bodies as indicated by arrowheads. Scale bar, 10 μm. INL, inner nuclear layer; ONL, outer nuclear layer; ROS, rod outer segment. (C) Scatterplot of detected photoreceptor cells with aberrant rhodopsin localization in 6 dpf zebrafish obtained by manual counting and plotted per counted eye (n = 12–14). Horizonal bars depict the mean number of photoreceptor cells with aberrant rhodopsin localization within a genotype. The number of cells with aberrant rhodopsin localization is significantly lower in wild-type, *ush2a*^*Δexon30-31*^, and *ush2a*^*Δexon39-40*^ retinas as compared with *ush2a*^*rmc1*^ retinas. Data were analyzed using one-way ANOVA followed by Tukey’s multiple comparison test. ∗p ≤ 0.05. ∗∗p ≤ 0.01.

Loss of usherin function was previously shown to lead to defects in rhodopsin transport from the inner segment toward the outer segment of photoreceptors (Figure 5A).^23^ We therefore investigated whether excision of *ush2a* exons 30–31 or exons 39–40 allows for normal rhodopsin trafficking. In the retinas of 6 dpf wild-type larvae, rhodopsin is localized to the photoreceptor outer segments (Figure 5B). In line with Toms and co-workers, occasionally photoreceptors in wild-type larvae were detected in which rhodopsin was partially localized to the inner segments.^23^ The amount of photoreceptor cells per retinal section with aberrant rhodopsin localization was significantly increased in retinas of *ush2a*^*rmc1*^ larvae (16.25 ± 8.96) as compared with wild-type controls (7.82 ± 8.54) (p = 0.03). Quantification revealed a significant reduction of the numbers of photoreceptor cells with aberrant rhodopsin localization in *ush2a*^*Δexon30-31*^ larvae (7.04 ± 6.88) and *ush2a*^*Δexon39-40*^ larvae (6.46 ± 4.49) as compared with *ush2a*^*rmc1*^ larvae (16.25 ± 8.96) (p = 0.02 [*ush2a*^*Δexon30-31*^] and p = 0.01 [*ush2a*^*Δexon39-40*^]), while those numbers were comparable in wild-type larvae (7.82 ± 8.54) (p = 0.99 [*ush2a*^*Δexon30-31*^] and p = 0.96 [*ush2a*^*Δexon39-40*^]), (Figure 5C). This suggests that usherin^Δexon30-31^ and usherin^Δexon39-40^ are able to support normal ciliary trafficking of rhodopsin to the photoreceptor outer segment.

### Design of splice-modulating ASOs

Based on the ability of usherin^Δexon30-31^ and usherin^Δexon39-40^ to properly localize in photoreceptors, to keep the composition of the USH2 protein complex intact and to support rhodopsin trafficking, we aimed to translate these findings into an ASO-based exon skipping approach for future use in humans. For this, ASOs specifically designed to induce skipping of our combined target exons from *USH2A* pre-mRNA were validated *in vitro*. For each exon of interest, multiple ASOs were designed to target either the intron-exon boundaries, or exonic splicing enhancer motifs within the exons (Figure S1 and Table S3). With the use of *in silico* analyses, parameters for (lack of) secondary structure formation, thermodynamic properties, and sequence selectivity were taken into account to minimize potential off-target effects. As a non-binding control, mismatch ASOs (mmASO) were used that contained four mismatches with the target sequence (Table S3). All ASOs contained 2′-O-(2-methoxyethyl) modified ribose groups and a fully phosphorothioated backbone.

To swiftly identify the most potent ASO for each of the target exons, the designed ASOs were co-transfected with the appropriate minigene splice vector in HEK293T cells at a 250-nM concentration, and screened for their potential to induce skipping of the target exon (Figure S3). Because the genomic region spanning *USH2A* exon 30 and exon 31 (∼22 kb) exceeds the practical limitations of Gateway cloning technology, individual vectors with either exon 30 and flanking sequences, or exon 31 and flanking sequences, were used in these experiments. The genomic region encompassing exons 39 and 40 spans around 0.8 kb and was cloned within a single minigene splice vector. For exons 31, 39, and 40, at least one ASO had high exon skipping potential. None of the individual ASOs designed to induce exon 30 skipping was shown to be effective. Simultaneous transfection of two ASOs directed against different parts of exon 30, resulted in a satisfactory level of exon skipping (Figure S4). ASO sequences with the highest observed exon skipping potential and sequences of the corresponding mismatch ASOs are listed in Table 1.

**Table 1:** ASO sequences

Antisense oligonucleotide	Sequence (5’>3′)
ASO_30A	CACUUUGUGGAGCUGUGAAGG
mmASO_30A	CACUUUGUAUAACUGUUAAGG
ASO_30E	GCUGUAUCCAUUUAAGCUGCG
mmASO_30E	GCUAUAUCCAUUUAAAAUACG
ASO_31B	CUUCUUGUGGAGUAGAGAUGUU
mmASO_31B	CUUAUUGUGUAUUAAAGAUGUU
ASO_39D	CUGGAGUUGGUAUCUGGGA
mmASO_39D	CUGAAUUUGAUAUCUGAGA
ASO_40A	UAGCUUAACGAUGCAGAAGGAUU
mmASO_40A	UAGAUUAAUGAUGCAUAAGUAUU

ASO, antisense oligonucleotide; mm, mismatch. Mismatches with the target sequence are underlined.

### ASOs induce a concentration-dependent increase of exon skipping in minigene splice assays

**Figure 6:**
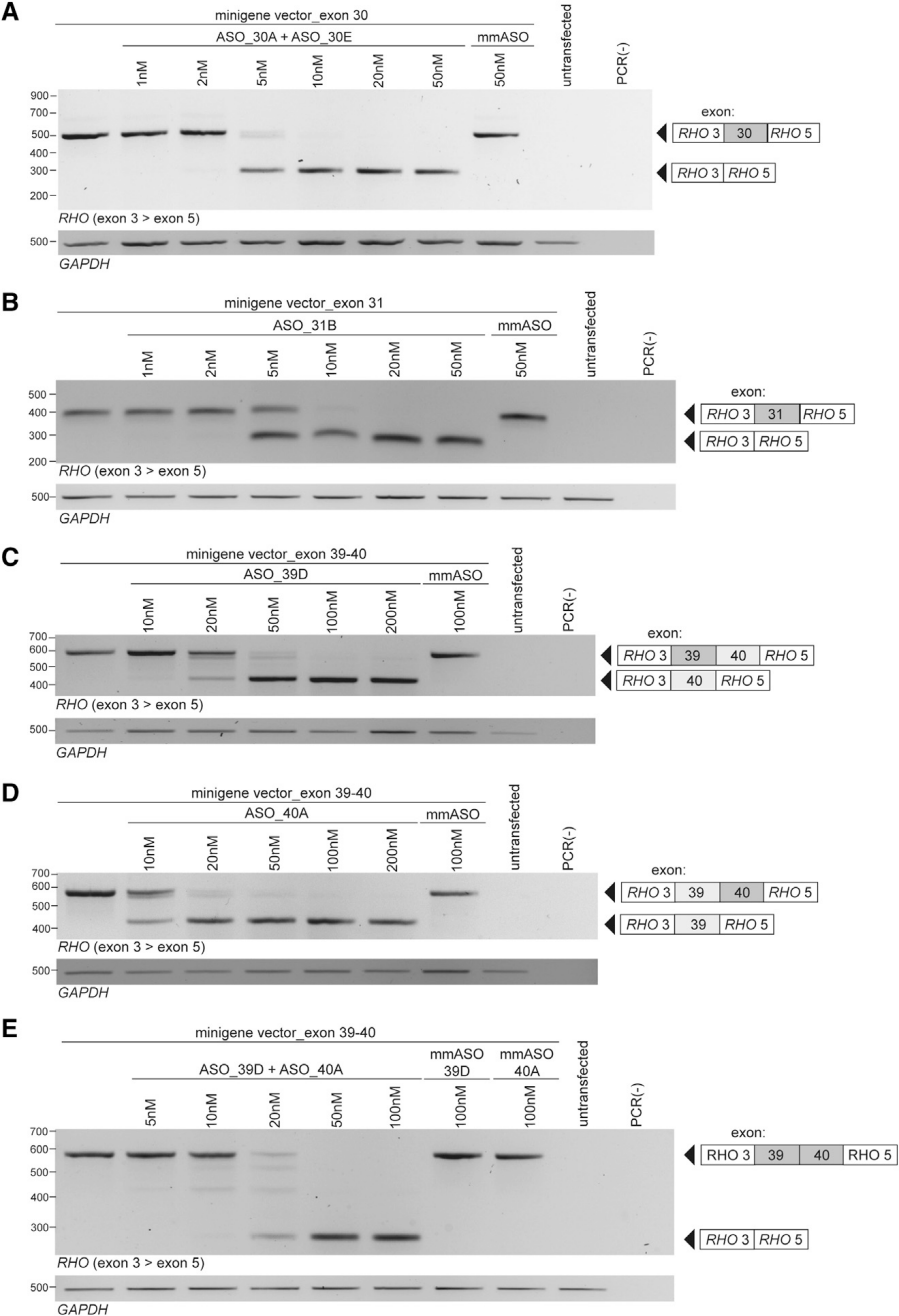
ASOs induce a dose-dependent skipping of *USH2A* target exons HEK293T cells are co-transfected with the exons 30–31 minigene splice vector and different concentrations of ASOs targeting (A) exon 30 and (B) exon 31 or with the exons 39–40 minigene splice vector and different concentrations of ASOs targeting (C) exon 39, (D) exon 40, or (E) exons 39 and 40. Each transfection resulted in a concentration-dependent increase in transcripts without the targeted exon(s). Amplicons with and without the targeted exon(s) are indicated adjacent to the gel image. For simultaneous transfection of multiple ASOs, the total ASO concentration is shown. *GAPDH* amplification is shown as a loading control. ASO, antisense oligonucleotide; mmASO, mismatch ASO; PCR(−), negative PCR control.

After the selection of the ASOs with the highest exon skipping potential from the initial screening, we lowered ASO concentrations in order to provide dose-ranging proof-of-concept for ASO-induced exon skipping. As shown in Figure 6, for all (combinations of) ASOs we observed a dose-dependent increase in the level of transcripts in which the target exons were skipped. The minimum effective dose for the ASOs targeting exons 30 and 31 is 5 nM, with full exon skipping achieved after treatment with 10 nM ASO. The minimum effective dose for the ASOs targeting exons 39 and 40 is 10–20 nM, with full exon skipping achieved after treatment with 100 nM ASO. As a control, we co-transfected the minigene splice vectors with a target-specific mmASO. These mmASOs were similar to the identified ASOs showing the highest exon skipping potential albeit with four mismatches to the target sequence. In none of the cases, and not even at the highest doses that were used, the mmASO was able to induce splice modulation, demonstrating the sequence specificity of the results. By co-transfecting the exons 39–40 minigene splice vector with the ASOs targeting exons 39 and 40, dual exon skipping could already be achieved at a total concentration of 20 nM ASO (10 nM ASO_39D and 10 nM ASO_40A) (Figure 6E). All exon–exon boundaries of the amplicons were sequence verified.

### ASOs induce dual exon skipping in WERI-RB1 cells

**Figure 7:**
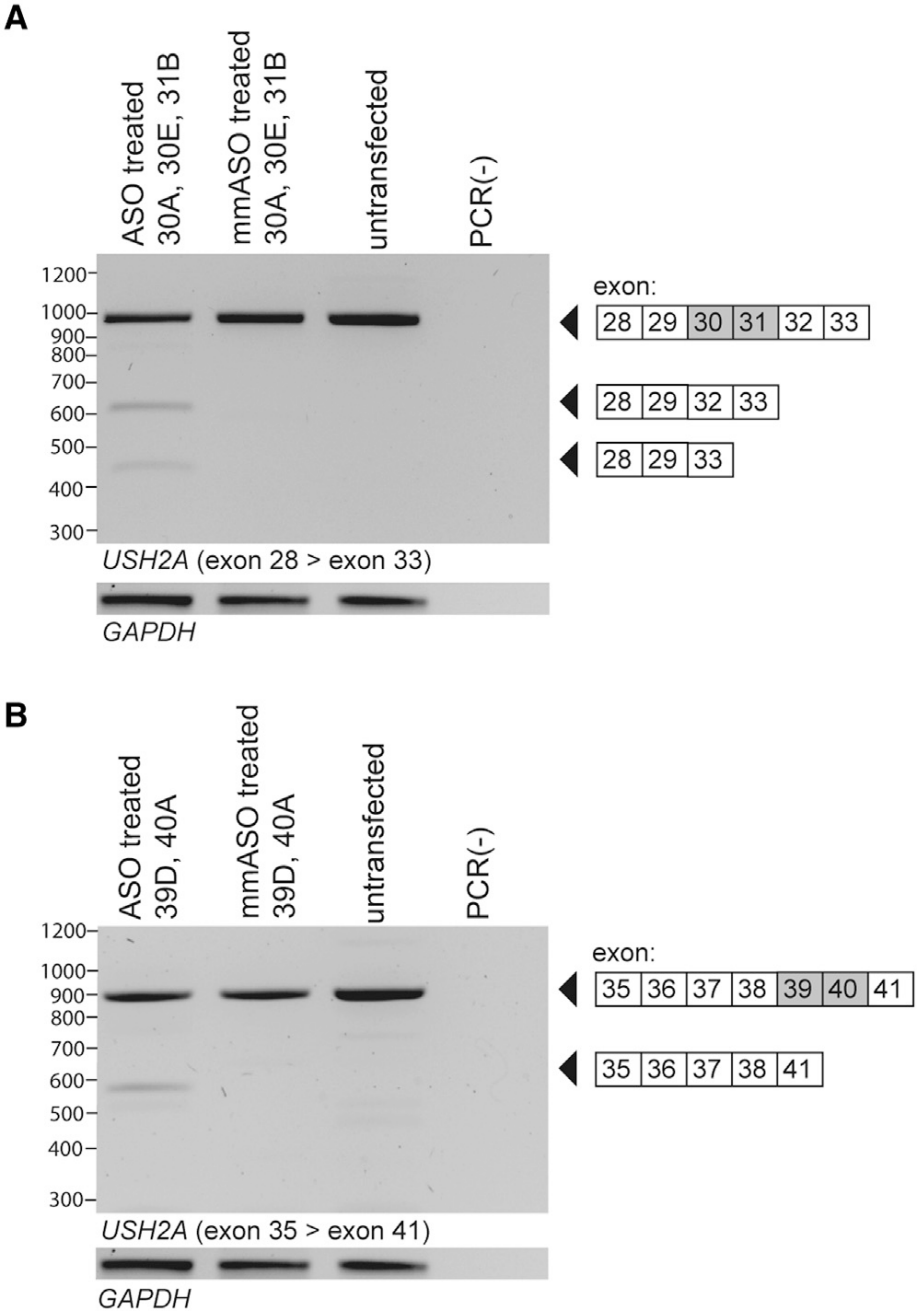
Validation of ASO-induced dual exon skipping for *USH2A* exons 30–31 and *USH2A* exons 39–40 in WERI-Rb-1 cells Co-transfection of WERI-Rb-1 cells with ASOs targeting either (A) exons 30 and 31 or (B) exons 39 and 40, resulted in the combined skipping of the exons of interest. Co-transfection of WERI-Rb-1 cells with ASOs targeting exons 30 and 31 also resulted in additional skipping of exon 32. In WERI-Rb-1 cells treated with ASOs targeting exons 39 and 40, no detectable amounts of alternatively spliced transcripts different from untransfected samples were present. *GAPDH* amplification is shown as a loading control. ASO, antisense oligonucleotide; mmASO, mismatch ASO; PCR(−), negative PCR control.

The retinoblastoma-derived WERI-Rb-1 cell line endogenously expressing *USH2A* was used to evaluate the potency for dual exon skipping of the identified ASOs.^24^ *USH2A* transcripts were analyzed by RT-PCR using primers in exon 28 and exon 33 (exons 30–31 skipping) or exon 35 and exon 41 (exons 39–40 skipping). Co-transfection of WERI-Rb-1 cells with selected ASOs in a 1:1 ratio for each target exon resulted in the anticipated effect of dual exon skipping (Figure 7). To induce skipping of exons 30 and 31, the combined ASOs targeting exon 30 were used at an equal amount as the ASO targeting exon 31 (25 nM ASO_30A, 25 nM ASO_30E, and 50 nM ASO_31B). Co-transfection of WERI-Rb-1 cells with these ASOs also resulted in a faint smaller splice product, which, after Sanger sequencing analysis, was identified as a transcript in which not only the target exons were lacking, but also exon 32. Unlike for exons 30–31, the combined transfection of ASOs directed against exons 39–40 (100 nM ASO_39D, 100 nM ASO_40A) did not result in detectable amounts of alternatively spliced transcripts additional to the transcript with the intended skipping of exons 39 and 40.

## Discussion

In this study, we explored the therapeutic potential of skipping combinations of two exons that together encode exactly one single protein domain. This approach may serve as a future paradigm to develop treatments aimed at slowing down or halting the progression of photoreceptor degeneration in *USH2A*-associated RP. Genomic excision of *USH2A* exons 30–31 and 39–40 indeed does not affect usherin expression and localization in the zebrafish retina, and does not interfere with rhodopsin localization. To translate this concept into a future use in patients, we developed and *in vitro*-validated a series of ASOs that induce the efficient skipping of the target exons from pre-mRNA, therewith offering an excellent basis for the further development of a dual exon skipping therapy for patients suffering from RP caused by mutations in *USH2A* exons 30, 31, 39, or 40.

Exon skipping therapies have been reported for many inherited disorders. For *USH2A*, we and others have previously shown that skipping of exon 13 can restore usherin function, and an ASO-based exon 13 skipping therapy (Ultevursen/QR-421a) has been evaluated in a phase 1/2 clinical trial (Trial # NCT03780257).^16^^,^^25^ Since two frequent founder mutations have been reported in *USH2A* exon 13, Ultevursen may already offer a treatment for about 35% of all patients with *USH2A*-associated RP.^13^^,^^16^ While an additional 26 of the 72 exons of *USH2A* can be skipped without disturbing the open reading frame, most exons do not encode a complete protein domain, and skipping of the individual exons is therefore unlikely to be therapeutic. To overcome this limitation, we employed a “domain-oriented” approach in which we searched for combinations of exons that are suitable for in-frame exon skipping, together encode a single protein domain and contain multiple uniq\eqalignno{}ue loss-of- function mutations. The combinations of exons 30–31 and exons 39–40 both meet these criteria, and inspired us to further investigate the functionality of usherin^Δexon30-31^ and usherin^Δexon39-40^.

Both in humans and zebrafish, *USH2A* exons 30–31 and exons 39–40 together encode a single FN3 domain within a series of 13 FN3 domains. Our studies in zebrafish confirm that the removal of either of these FN3 domains does not affect the expression and subcellular localization of usherin. Similar to native usherin, usherin^Δexon30-31^ and usherin^Δexon39-40^ are able to scaffold the USH2 protein network at the photoreceptor periciliary membrane, as visualized by the Adgrv1 immunoreactivity in this region. This periciliary USH2 protein complex is hypothesized to play a role in the transfer of cargo, such as rhodopsin, from the inner segment to the outer segment.^5^ It is well accepted that the accumulation of outer segment proteins in the inner segment, for example to due to ciliary trafficking defects, can lead to photoreceptor degeneration through the activation of cell stress pathways and ectopic phototransduction.^26^^,^^27^ Indeed, defective rhodopsin trafficking, activation of autophagy, and photoreceptor apoptosis were previously observed in *ush2a* mutant zebrafish and support the notion that this process lies at the origin of photoreceptor cell death in *USH2A*-associated RP.^6^^,^^23^ The observation that the number of cells with rhodopsin accumulation in the photoreceptor cell body is similar in wild-types, *ush2a*^*Δexon30-31*^ and *ush2a*^*Δexon39-40*^ zebrafish, but elevated in *ush2a*^*rmc1*^ zebrafish, therefore suggests that our exon-excision approach can prevent or delay photoreceptor degeneration.

ASO-mediated skipping of native exons has emerged as a promising therapeutic strategy for several inherited disorders that result from mutations in large genes encoding (structural) proteins that contain a series of repetitive protein domains.^28^^,^^29^^,^^30^^,^^31^ However, only a few studies investigated the therapeutic potential of dual exon skipping. In a study by Aartsma-Rus and co-workers, ASO-mediated co-skipping of *DMD* exons 43 and 44 resulted in the restoration of dystrophin levels in patient-derived myotubes.^18^ In addition, Rutten and co-workers showed the successful dual skipping of *NOTCH3* exons 2–3 and exons 4–5 in vascular smooth muscle cells derived from a patient with CADASIL.^19^ The previously targeted *USH2A* exon 13 and *NOTCH3* exons encode one or more (parts of) EGF domains, resulting in the formation of a functionally related hybrid EGF or EGF-like fusion domain.^16^^,^^19^ To our notice, we are first to employ a domain-oriented exon skipping approach as the aforementioned studies primarily selected the target exons based on mutation hotspots. Usherin is predicted to contain a total of 28 FN3 domains, which all show a strong structural conservation having a minimum of seven beta strands connected with loops that can vary between domains in terms of length and folding. As the *USH2A* regions that we aimed to skip encode exactly one FN3 domain, the potential risks associated with the formation of a fusion domain, in which parts of different domains are fused, are avoided. In fact, both usherin^Δexon30-31^ and usherin^Δexon39-40^ have the same domain architecture, which leads us to speculate that any of the 13 FN3 domains in the middle section of usherin could be targeted for exon skipping therapy as long as they are precisely encoded by one or more in-frame exons. Furthermore, it would be worthwhile investigating the minimal structural requirements of usherin in order to be functional. So far, it remains to be determined whether this approach could be successfully applied to any FN3 domain or combination of FN3 domains within usherin, and whether this approach could also be applied to other proteins containing a series of FN3 or other domains in general. For *EYS*-associated RP, the excision of multiple exons encoding a repetitive combination of one laminin G and two EGF-lam domains from the zebrafish genome did not result in the expression of a shortened Eys protein in photoreceptors.^32^ Generating comprehensive sets of structural models based on 3D homology modeling enables a better prediction of (combinations of) exons that would be potentially eligible as targets in exon skipping strategies, not only for *USH2A*, but also for other large genes encoding proteins with a repetitive domain architecture.

Following therapeutic proof-of-concept in zebrafish, we designed and validated ASOs targeting *USH2A* exons 30–31 and exons 39–40 as candidate molecules to induce skipping of the target exons in human cells. In our minigene splice assay, the identified ASOs were able to induce skipping of the individual exons at much lower concentrations compared with those previously developed to correct aberrant pre-mRNA splicing of a cryptic exon in *USH2A* intron 40.^15^ To the best of our knowledge, retinoblastoma cell lines such as WERI-Rb-1 are the only cell lines with robust *USH2A* expression levels.^24^ We used these cells and evaluated the potency for dual exon skipping of ASO combinations on the endogenous full-length *USH2A* transcript. Indeed, we were able to induce dual exon skipping of exons 30–31 and exons 39–40 in WERI-Rb-1 cells. Although we increased the ASO concentrations far beyond the optimum observed in our minigene assay, the observed levels of co-skipping remained minimal. One potential explanation for this phenomenon is a poor transfection efficiency, as WERI-Rb-1 cells grow in grape-like clusters that are difficult to transfect. Alternatively, a slow turnover of *USH2A* transcripts results in a constant population of completely spliced mRNA molecules. The benefit of investigating the effect of splice modulation on native *USH2A* transcripts is the ability to visualize the skipping of multiple exons, both intended and unintended. While we observed perfect dual exon skipping for both exons 30–31 and exons 39–40 upon ASO treatment of WERI-Rb-1 cells, we also noticed the unintended co-skipping of exon 32 upon co-transfection of ASOs targeting exons 30 and 31. The additional skipping of exon 32 retains the open reading frame, but is predicted to disrupt the neighboring FN3 domain, most likely with a detrimental effect on the overall protein structure. However, it is important to realize that WERI-Rb-1 cells are obtained from a retinal tumor, and pre-mRNA splicing might not completely mimic the events that occur in healthy photoreceptors. For example, WERI-Rb-1 cells display prominent alternative splicing events for *USH2A* exons 12–14 that are not recapitulated in iPSC-derived photoreceptor progenitor cells.^16^ Second, there is a significant proportion of transcripts with perfect skipping of exons 30–31 that could already be sufficient to restore retinal function. As such, it is important to further investigate this in a cellular model with a more relevant genetic and transcriptional profile, such as iPSC-derived retinal organoids.

The CRISPR-Cas9-mediated exon-excision approach, as employed in our zebrafish model, physically removes the target region at the level of genomic DNA, and a single treatment would already result in a permanent effect. While many technical and ethical issues accompanying CRISPR-Cas9-based genome editing hamper the application in patients, such an approach may both resolve the issue with exon 32, and provide patients with a treatment that requires only a single administration.^33^ However, key advantages of splice modulation using ASOs are the anticipated safety of targeting the transcriptome rather than the genome, and the recent clinical successes obtained with molecules that have a similar chemistry and functionality.^34^^,^^35^^,^^36^ To achieve long-term effective splice modulation in patients, it is possible to use a viral vector-based system to deliver (combinations of) ASOs to the retina (#NCT04240315).^37^^,^^38^^,^^39^^,^^40^ While synthetic ASOs benefit from the ease of simple rational design, and limited off-target and inflammatory effects, virally delivered ASOs may encounter challenges such as the natural presence of neutralizing antibodies against the anticipated viral vectors, virus-induced immunogenicity, unwanted genomic integration, and the inability to re-dose.^35^^,^^41^

With the successful experimental treatment of patients with Ultevursen with the aim to halt or slow down the progression of *USH2A*-associated RP, the first ASO-based treatment for this condition seems within reach. Since the majority of patients do not have mutations in exon 13, we now expanded the exon skipping approach by exploring the potential of dual exon skipping after targeting carefully selected combinations of in-frame exons, using zebrafish as a functional model. Although inter-species differences in usherin may exist, the translational value of zebrafish models in retinal research has been repeatedly proven.^6^^,^^16^^,^^20^^,^^42^^,^^43^ In fact, the functional proof-of-concept for the ASO-induced skipping of *USH2A* exon 13 was solely based on experiments performed in zebrafish, when QR-421a entered the clinical phase. Although a follow-up analysis of usherin expression and function after ASO-mediated skipping of exons 30–31 and exons 39–40 in more advanced human disease models will offer valuable insight in the feasibly of our approach in the human retina, all evidence suggests that a single FN3 domain can be removed from usherin without severely hampering its function. As such, our work offers a highly promising outlook for patients with mutations in *USH2A* exons 30, 31, 39, and 40. Extrapolation of our data to other (multiple) exons encoding FN3 domains can provide an even larger population of patients with *USH2A*-associated RP a prospect on retaining vision.

## Materials and methods

### Zebrafish ethics, maintenance, and husbandry

Animal experiments were conducted in accordance with the Dutch guidelines for the care and use of laboratory animals (Wet op de Dierproeven 1996) and European regulations (Directive 86/609/EEC), as approved by the Dutch Ethics committee of the Central Committee Animal Experimentation (Centrale Commissie Dierproeven [CCD]; application number AVD103002017945). We used wild-type Tupfel Longfin (TL) zebrafish to generate *ush2a*^*Δexon30-31*^ (c.5924–114_6223 + 419delinsTT; p.V1975_E2074del) and *ush2a*^*Δexon39-40*^ (c.7346–848_7635 + 5863del; p.A2449_R2540delinsG) therapeutic models, deposited as *ush2a*^*rmc21*^ and *ush2a*^*rmc17*^ in ZFIN, respectively (ENSDART00000086201.5). In addition, we employed the previously described *ush2a*^rmc1^ mutant (c.2337_2342delinsAC; p.Cys780GlnfsTer32).^6^ Zebrafish were maintained and raised according to standard methods.^44^ Both adult and larval zebrafish were kept at a light-dark regimen of 14 h of light and 10 h of darkness. Adult zebrafish were daily fed twice with Gemma Micro 300 dry pellets (#13177, Zebcare, Nederweert, The Netherlands) at ∼5% body weight and once with artemia. Embryos were obtained from natural spawning.

### Multiple sequence alignment

A multiple sequence alignment of human usherin (ENSP00000305941_3) and zebrafish usherin (ENSDARP00000080636_3) was generated using AlignX in the Vector NTI software package (Vector NTI Advance 11).

### *In silico* modeling of the effect of *USH2A* exons 30–31 and 39–40 skipping on FN3 protein domain structure

The amino acid sequence of human usherin was selected from the UniProtKB database (https://www.uniprot.org/; acc. #O75445) and used as a template to model the effect of the skipping of *USH2A* exons 30–31 and 39–40 on the 3D protein domain structure of usherin. The effect of exons 30–31 skipping on the protein domain structure was modeled in a chain of one LamG domain followed by three consecutive FN3 domains. For prediction of the wild-type protein structure LamG(2), FN3(5), FN3(6), and FN3(7) were included, of which FN3(6) is encoded by exons 30–31. The structural model of usherin^Δexon30-31^ included LamG(2), FN3(5), FN3(7), and FN3(8). Effect of exons 39–40 skipping on the usherin structure was modeled in a chain of three consecutive fibronectin type III (FN3) domains. For prediction of the wild-type protein structure FN3(10), FN3(11), and FN3(12) were included, of which FN3(11) is encoded by exons 39–40. The model of usherin^Δexon39-40^ includes FN3(10), FN3(12), and FN3(13). The AlphaFold2 (https://colab.research.google.com/github/sokrypton/ColabFold/blob/main/AlphaFold2.ipynb) modeling script was used to generate the structural models of both wild-type and usherin^Δexon^ proteins based on PSI-BLAST alignments produced with sequences of PDB full-chain representatives (<70% sequence identity) (PDB70) by employing standard parameters.^45^

### CRISPR-Cas9 genome-editing design

Target sites for sgRNAs to cleave in introns 29 and 31, and in 38 and 40 of zebrafish *ush2a* (NCBI accession XM_009293147.3) were identified with the online web tool CHOPCHOP (https://chopchop.cbu.uib.no/).^46^ sgRNAs for which no off-target sites were predicted and which had the highest predicted efficiency score were selected for synthesis.^47^ Synthesis of sgRNAs was performed as described previously.^48^ In brief, templates for *in vitro* sgRNA transcription were generated by annealing a constant oligonucleotide encoding the reverse complement of the tracrRNA tail to a target-specific oligonucleotide containing the T7 promoter sequence (5′-20-base target sequence, and a region (5′-GTTTTAGAGCTAGAAATAGCAAG-3′) complementary to the constant oligonucleotide. Phusion High-Fidelity DNA Polymerase (#M0530L, New England Biolabs, Ipswich, MA, USA) was used to fill the single-stranded DNA overhang after which the template was purified using the GenElute PCR clean-up kit (#NA1020-1KT, Sigma-Aldrich, St. Louis, MO, USA). The template was used for the *in vitro* transcription of the sgRNAs using the T7 MEGAshortscript Kit (#AM1354, Thermo Fisher Scientific, Waltham, MA, USA). Obtained transcripts were purified using the MEGAclear Transcription Clean-Up Kit (#AM1908, Thermo Fisher Scientific, Waltham, MA, USA). Target-specific oligonucleotides used for sgRNA synthesis are listed in Table S1.

### Microinjections

For the generation of the *ush2a*^*Δexon30-31*^ and *ush2a*^*Δexon39-40*^ zebrafish (deposited as *ush2a*^*rmc21*^ and *ush2a*^*rmc17*^ in ZFIN, respectively), the 5′ sgRNA, 3′ sgRNA and commercial Alt-R S.p. Cas9 Nuclease V3 (#1081059, IDT, Newark, NJ, USA) were co-injected. To avoid preferential *in vivo* binding of Cas9 to either sgRNA, individual sgRNA-Cas9 complexes were prepared and combined prior to injection. For this, the individual mixtures were incubated at 37°C for 5 min after which they were combined. The final injection mix contained 80 ng/μL 3′ sgRNA, 80 ng/μL 5′ sgRNA, 800 ng/μL Cas9 protein, 0.3 M KCl, and 0.1% phenol red. Injection needles (#TW120F-3, World Precision Instruments, Friedberg, Germany) were prepared using a micropipette puller (Model P-97, Sutter Instrument Company, Novato, CA, USA). A minimum of 100 wild-type zebrafish embryos were collected after natural spawning and injected at the single-cell stage with 1 nL of injection mixture using a Pneumatic PicoPump (#SYS-PV820, World Precision Instruments, Friedberg, Germany). After injection, embryos were raised at 28.5°C in E3 embryo medium (5 mM NaCl, 0.17 mM KCl, 0.33 mM CaCl2, and 0.33 mM MgSO4) supplemented with 0.1% (v/v) methylene blue. At 1 dpf, 25% of the injected embryos were analyzed for the presence of the anticipated exon deletion using genomic PCR analysis. The remainder of the injected embryos were raised to adulthood.

### Genotyping

Genomic DNA was extracted from whole larvae (1 dpf) or caudal fin tissue from adult zebrafish. Tissue was lysed in 25 μL (larvae) or 75 μL (fin tissue) lysis buffer (40 mM NaOH, 0.2 mM EDTA) at 95°C for 20 min. The lysed samples were neutralized with 0.1 lysis volume of 1M TRIS-HCl (pH 7.5) and diluted 10 times with Milli-Q water. One microliter of diluted sample was used as a template in PCR reactions to amplify the zebrafish *ush2a*^*Δexon30-31*^ allele, the zebrafish *ush2a*^*Δexon39-40*^ allele, and the corresponding wild-type zebrafish *ush2a* alleles. For PCR analysis, the Q5 High-Fidelity DNA Polymerase kit (#M0491L; New England Biolabs, Ipswich, MA, USA) was employed. All primer sequences are listed in Table S2. The presence or absence of the *ush2a*^*Δexon30-31*^ allele and the *ush2a*^*Δexon39-40*^ allele was confirmed by Sanger sequencing.

### Immunohistochemistry, histology, and quantification of fluorescent signal intensity

Zebrafish *ush2a*^*Δexon30-31*^ and *ush2a*^*Δexon39-40*^, *ush2a*^rmc1^, and strain-matched wild-type larvae (5 dpf) were cryoprotected with 10% sucrose in PBS for 10 min prior to embedding in OCT compound (Tissue-Tek, #4583, Sakura, Alphen aan den Rijn, The Netherlands). After embedding, samples were snap frozen in liquid nitrogen-cooled isopentane and sectioned following standard protocols. Cryosections (7-μm thickness along the lens/optic nerve axis) were rinsed with PBS, permeabilized for 20 min with 0.01% Tween 20 in PBS and blocked for 30 min with blocking buffer (10% normal goat serum and 2% bovine serum albumin in PBS). Antibodies diluted in blocking buffer were incubated overnight at 4°C. Secondary antibodies were also diluted in blocking buffer and incubated together with DAPI (1:8,000; D1306; Molecular Probes, Eugene, OR, USA) for 1 h. Sections were post fixed with 4% paraformaldehyde for 10 min and mounted with Prolong Gold Anti-fade (P36930; Molecular Probes, Eugene, OR, USA). The following primary antibodies and dilutions were used: rabbit anti-usherin (1:500; #27640002, Novus Biologicals, Centennial, CO, USA), mouse anti-centrin (1:500; #04–1624, Millipore, Burlington, MA, USA), and rabbit anti-Adgrv1 (1:100; #DZ41033, Boster Bio, Pleasanton, CA, USA). Secondary antibodies (Alexa Fluor 568 goat anti-rabbit (#A11011, Thermo Fisher Scientific, Waltham, MA, USA), Alexa Fluor 647 goat anti-mouse (#A21237, Thermo Fisher Scientific, Waltham, MA, USA) and Alexa Fluor 488 goat anti-mouse (#A11029, Thermo Fisher Scientific, Waltham, MA, USA)) were used in a 1:800 dilution. Images were taken using a Zeiss Axio Imager fluorescence microscope equipped with an AxioCam MRC5 camera (Zeiss, Jena, Germany). Quantification of the fluorescent signal intensity of anti-usherin and anti-Adgrv1 immunoreactivity was performed using Fiji version (v.) 1.47 software as described previously.^16^^,^^49^ Upon identification of the areas of the connecting cilia, the maximum and minimum gray value of usherin or Adgrv1 immunofluorescence in those areas was measured. The difference between those values was calculated for each individual photoreceptor cell after which the mean difference per retina was plotted. All data were analyzed using one-way ANOVA followed by Tukey’s multiple comparison test and described as mean ± SD. Statistical significance was set at p < 0.05.

To assess rhodopsin localization in the larval retina, larvae (6 dpf) from homozygous *ush2a*^*Δexon30-31*^, *ush2a*^*Δexon39-40*^, *ush2a*^rmc1^ and strain-matched wild-type controls were sampled 100 min post light onset. Larvae were fixed in darkness overnight at 4°C using 4% paraformaldehyde, dehydrated using methanol series with an ascending concentration, transferred to 100% methanol for an overnight incubation followed by storage at −20°C. Upon embedding, larvae were rehydrated in descending methanol series to 0.1% PBS-Tween-20. Afterward, larvae were cryoprotected with 10% sucrose in 0.1% PBS-Tween-20 for 15 min, followed by an incubation in 30% sucrose in 0.1% PBS-Tween-20 for 1 h at room temperature. Larvae were then embedded, snap frozen and sectioned as described above. Cryosections were rinsed with PBS, permeabilized for 2 min with 0.1% Tween 20 in PBS and, immersed in 10mM Sodium Citrate at pH 8.5 and heated for 1 min at 121°C in the autoclave. Cryosections were subsequently washed in 0.1% Tween 20 in PBS and blocked for 1 h with blocking buffer (10% non-fat dry milk and 0.1% Tween 20 in PBS). Primary antibody (mouse anti-rhodopsin, 1:4000, #NBP2-59690, Novus Biologicals, Centennial, CO, USA) diluted in blocking buffer was incubated overnight at 4°C. Secondary antibody (Alexa Fluor 488 goat anti-mouse, 1:800, #A11029, Thermo Fisher Scientific, Waltham, MA, USA) was also diluted in blocking buffer and incubated together with DAPI (1:8000; #D1306; Thermo Fisher, Waltham, MA, USA) for 1.5 h. Sections were mounted and images were taken as described above. Rhodopsin levels were quantified by manual counting. For this, all pictures were taken using the same settings after which the mislocalization spots in the region of interest (outer nuclear layer), blinded and analyzed independently by two individuals. For all pictures mean counts were calculated and analyzed using a one-way ANOVA followed by Tukey’s multiple comparison test. Statistical significance was set at p < 0.05 and data are described as mean ± SD.

### Antisense oligonucleotides

The sequences of *USH2A* exons 30, 31, 39, and 40 (with 50 bp upstream and downstream flanking intronic sequence) were analyzed to identify potential ASO target sites as described previously (Figure S1).^50^ Briefly, the presence of exonic splice enhancer motifs was assessed using the “Human Splicing Finder” website (http://www.umd.be/HSF3/; Date accessed: December 16, 2020) and RNA structure and free energy predictions were performed using freely available database tools (http://www.unafold.org/; http://rna.urmc.rochester.edu/RNAstructureWeb/index.html). ASOs were designed to have a Tm ≥ 48°C, a GC content between 40% and 60% and a length of 17–23 nt. Subsequently, for each targeted exon, the 2–6 most optimal ASOs were purchased from Eurogentec (Liège, Belgium) containing 2′-O-(2-methoxyethyl) modified ribose groups and a fully phosphorothioated backbone. The matching control ASOs all contain four mismatches relative to the target sequence. All ASOs were dissolved in PBS before use. ASO sequences are listed in Table S3.

### Minigene splice vectors

The genomic region containing human *USH2A* exons 39 and 40, exon 30, or exon 31, together with 300–1,000 base pairs (bp) of flanking up- and downstream intronic sequence, was cloned into a pDONR201 vector using Gateway cloning technology. Primer sequences are listed in Table S2. The complete inserts of the donor vectors were sequence verified and subsequently cloned into the pCI-Neo-Rho destination vector, which enables the expression of the fragment of interest flanked by two rhodopsin exons.^15^ This resulted in three minigene splice vectors, either containing human exon 39 and 40, exon 30, or exon 31 (Figure S2).

### Cell culture

HEK293T cells were cultured in Dulbecco’s modified Eagle’s medium (DMEM) (#D0819, Sigma-Aldrich, St. Louis, MO, USA) supplemented with 10% (v/v) fetal bovine serum (#F7524, Sigma-Aldrich, St. Louis, MO, USA), 1% penicillin-streptomycin (#P4333, Sigma-Aldrich, St. Louis, MO, USA) and 1% sodium pyruvate (#S8636, Sigma-Aldrich, St. Louis, MO, USA). Cells were passaged twice per week upon standard trypsinization (#DF0152-15-9, Thermo Fisher Scientific, Waltham, MA, USA). WERI-Rb-1 cells were cultured in RPMI-1640 (#22409–015, Gibco Waltham, MA, USA) supplemented with 15% (v/v) fetal bovine serum (#F7524, Sigma-Aldrich, St. Louis, MO, USA), 2% HEPES (#H0887, Sigma-Aldrich, St. Louis, MO, USA), and 1% penicillin-streptomycin (#P4333, Sigma-Aldrich, St. Louis, MO, USA). Cells were cultured in suspension and maintained by replacement of the medium every 3 to 4 days.

### Transfection of ASOs and minigene splice vectors in HEK293T cells

HEK293T cells were seeded at a concentration of ∼0.2 × 10^5^ cells per well in a 24-well plate and grown for 24 h at 37°C in a total volume of 0.5 mL medium. Cells were (co-)transfected with 500 ng of the minigene splice vector and the indicated amount of ASO, calculated as the final concentration in the culture medium after ASO delivery. The transfection mixture furthermore contained 3 μL Fugene HD Transfection Reagent (#E2311, Promega, Madison, WI, USA), and was prepared in a final volume of 50 μL Opti-Mem (#31985–047, Gibco Waltham, MA, USA), according to manufacturer’s protocol. Two wells per condition were treated. After incubation for 24 h at 37°C, cells were washed once with PBS and harvested for RNA isolation.

### Transfection of ASOs in WERI-Rb-1 cells

Transfections were performed on adherend cells. For this purpose, all wells of a 12-well plate were coated with Poly-L-Lysine (#P4707, Sigma-Aldrich, Saint Louis, MO, USA) by adding 0.5 mL Poly-L-Lysine to each well. After a 90-min incubation at 37°C, Poly-L-Lysine was removed from the wells and wells were washed three times with PBS and air-dried for 30 min. Next, WERI-Rb-1 were seeded at a concentration of 1.0 × 10^6^ cells per well in a 12-well plate and incubated for 48 h at 37°C. Cells were subsequently transfected with the ASO of interest using Lipofectamine 2000 transfection reagent (#11668019, Thermo Fisher Scientific, Waltham, MA, USA) at a 2:1 (volume:weight) ratio between Lipofectamine 2000 and ASO. First, individual Lipofectamine 2000/Opti-MEM (50 μL) mixtures and an ASO/Opti-MEM (50 μL) mixtures were prepared. Both mixtures were individually incubated at room temperature for 5 min. Next, the ASO and Lipofectamine mixtures were mixed together and incubated at room temperature for an additional 10 min before being added to the cells. After a 24-h incubation at 37°C, cells were washed once with PBS and harvested for RNA isolation.

### RNA isolation and cDNA synthesis

Total RNA was isolated from transfected HEK293T cells or WERI-Rb-1 cells using the Nucleospin RNA II isolation kit (#740955.250, MACHEREY-NAGEL, Düren, Germany), according to the manufacturer’s protocol, whereas total RNA from zebrafish larvae (5 dpf) was extracted using the RNeasy Micro kit (#74004, Qiagen, Hilden, Germany). For cDNA synthesis from HEK293tRNA, the iScript cDNA synthesis kit (#1708891, Bio-Rad, Hercules, CA, USA) was used with 0.5 μg total RNA as input. From WERI-Rb-1 and zebrafish RNA, cDNA was synthesized from 0.1 to 0.3 μg total RNA using SuperScript IV Reverse Transcriptase (#18090010, Thermo Fisher Scientific, Waltham, MA, USA), combined with an oligo(dT)_12-18_ primer (#18418012, Thermo Fisher Scientific, Waltham, MA, USA), according to the manufacturer’s protocol.

### Transcript analysis

For the exon skipping experiments using the minigene splice vectors, the target region was amplified from the synthesized cDNA using Taq polymerase (M0491L, New England Biolabs, Ipswich, MA) and a forward primer and reverse primer located in exons 3 and 5 of the human *RHO* gene, respectively. For the experiments in WERI-Rb-1 cells and zebrafish larvae, the target region was amplified from the synthesized human or zebrafish cDNA using Q5 High-Fidelity DNA Polymerase (#M0491L, New England Biolabs, Ipswich, MA, USA). For the exon skipping experiments in HEK293T cells and WERI-Rb-1 cells, primers amplifying *GAPDH* using Taq polymerase (M0491L, New England Biolabs, Ipswich, MA) were employed as a control. All primer sequences are listed in Table S2. Amplified fragments were separated on a 1% agarose gel and sequence verified by Sanger sequencing.

## Data availability statement

Not applicable.

## Supplemental information

Document S1. Figures S1–S4 and Tables S1–S3

Document S2. Article plus supplemental information
