# Supplementary material for: A protein domain-oriented approach to expand the opportunities of therapeutic exon skipping for USH2A-associated retinitis pigmentosa
Source: Mol Ther Nucleic Acids. 2023 May 20;32:980–94. doi: 10.1016/j.omtn.2023.05.020 (PMC10258241; doi:10.1016/j.omtn.2023.05.020)
Supplement: Document S1. Figures S1–S4 and Tables S1–S3 [file mmc1.pdf]

OMTN, Volume *32*

Supplemental information

A protein domain-oriented approach to expand the opportunities of therapeutic exon skipping for *USH2A*-associated retinitis pigmentosa

Renske T.W. Schellens, Sanne Broekman, Theo Peters, Pam Graave, Lucija Malinar, Hanka Venselaar, Hannie Kremer, Erik De Vrieze, and Erwin Van Wijk

Table S1. Oligonucleotide sequences used for sgRNA synthesis.

Oligo Name	Sequence (5’ > 3’)
**constant oligo**	AAAAGCACCGACTCGGTGCCACTTTTTCAAGTTGATAACGGACTAGCCTTATTTTAACTTGCTATTTCTAGCTCTAAAAC
**target-specific oligo 5’ *ush2a*** $^{\Delta exon30\text{-}31}$	CCGCTAGC**TAATACGACTCACTATA***GGGTGAGAATCCAATTGGGA*GTTTTAGAGCTAGAAATAGCAAG
**target-specific oligo 3’ *ush2a*** $^{\Delta exon30\text{-}31}$	CCGCTAGC**TAATACGACTCACTATA***GGGCTAAAAAGAATTGATTG*GTTTTAGAGCTAGAAATAGCAAG
**target-specific oligo 5’ *ush2a*** $^{\Delta exon39\text{-}40}$	CCGCTAGC**TAATACGACTCACTATA***GGGACAGAAGCGAGGGTCTG*GTTTTAGAGCTAGAAATAGCAAG
**target-specific oligo 3’ *ush2a*** $^{\Delta exon39\text{-}40}$	CCGCTAGC**TAATACGACTCACTATA***GGGGTGATGACTTTGAGAAC*GTTTTAGAGCTAGAAATAGCAAG

T7 promoter sequence in bold. Gene specific region in italics. Overlapping regions of the constant and target-specific oligonucleotides are underlined.

Table S2. Primers used for RT-PCR analysis.

	Primer name	Primer sequence (5' > 3')
ush2a$^{\Delta exon30\text{-}31}$ **genotyping**	*ush2a* wild-type forward (1)	CTCCACAAGAGGGTAAAGTC
	ush2a wild-type reverse (1)*	TTGGACCGGCTTTATTTCTG
	ush2a Δexon30-31 forward	TTCACTTCATGCCATTCAGC
	ush2a Δexon30-31 reverse*	TTGGACCGGCTTTATTTCTG
ush2a$^{\Delta exon39\text{-}40}$ **genotyping**	*ush2a* wild-type forward (2)	TGGTCACTTTCCAACAAAACCT
	ush2a wild-type reverse (2)	CAGAAGACCATCTGGAGCTGT
	ush2a Δexon39-40 forward	TGCAATCCCTTCTTTTGTCA
	ush2a Δexon39-40 reverse	GTTTATTAGCAGTGCCAGGAGC
ush2a$^{\Delta exon30\text{-}31}$ **transcript analysis**	*ush2a* exon 28-33 forward	CCCTGCTCTGAACAAACACA
	ush2a exon 28-33 reverse	TGTTGGTACATCCTGCCTCA
ush2a$^{\Delta exon39\text{-}40}$ **transcript analysis**	*ush2a* exon 37-42 forward	ACCTACACAGTCCTCTTCGC
	ush2a exon 37-42 forward	GTGGTTCAGGCATTCGGATC
**Minigene splice vector *USH2A* exon 30**	*USH2A* intron 29-30 forward	GGGGACAAGTTTGTACAAAAAAGCAG<br>GCTTCTGCTTCAAAGGGGGTACATC
	USH2A intron 29-30 reverse	GGGGACCACTTTGTACAAGAAAGCTGG<br>GTCCATTCTTCATGGCACACCAC
**Minigene splice vector *USH2A* exon 31**	*USH2A* intron 30-31 forward	GGGGACAAGTTTGTACAAAAAAGCAG<br>GCTTCACTGGGGAGGGAAATTATGG
	USH2A intron 30-31 reverse	GGGGACCACTTTGTACAAGAAAGCTGG<br>GTCCCTGTGCTGCTGTTTAG
**Minigene splice vector *USH2A* exon 39-40**	*USH2A* intron 38-40 forward	GGGGACAAGTTTGTACAAAAAAGCAG<br>GCTTCAGGGCAGGAGAAGGGATAAC
	USH2A intron 38-40 reverse	GGGGACCACTTTGTACAAGAAAGCTGG<br>GTGATGCTGAAGGCTGTCTTTGG

**Minigene splice assay HEK293T cells**	*Rho* exon 3-5 forward	CGGAGGTCAACAACGAGTCT
	Rho exon 3-5 reverse	AGGTGTAGGGGATGGGAGAC
**Splice assay WERI-Rb-1 cells *USH2A*$^{\Delta exon30\text{-}31}$**	*ush2a* exon 28-33 forward	CCAGGGAAAAGAGCAGAGTG
	ush2a exon 28-33 reverse	GCATATGATCCTGGAAAAGTTC
**Splice assay WERI-Rb-1 cells *USH2A*$^{\Delta exon39\text{-}40}$**	*ush2a* exon 37-42 forward	GTCTCCTGGACTGAGCCTGA
	ush2a exon 37-42 reverse	CATCCAGAAGAATCGGAGGA
**Loading control**	*GAPDH* forward	ACCACAGTCCATGCCATCAC
	GAPDH reverse	TCCACCACCCTGTTGCTGTA

Gateway cloning tails underlined; * = primers are identical.

Table S3. Antisense oligonucleotide characteristics.

RNA oligonucleotide	Sequence (5'>3')	Length (nt)	GC content (%)
**ASO_30A**	CACUUUGUGGAGCUGUGAAGG	21	52
**ASO_30B**	AAUUCAGCACUGGCAGAGG	19	53
**ASO_30C**	CAGGUCACCUCAAUGCUGUA	20	50
**ASO_30D**	UGGCACACUUUGUGGAGCU	19	53
**ASO_30E**	GCUGUAUCCAUUUAAGCUGCG	19	53
**ASO_31A**	GUAGCAAGCCUGUCAAUAUGCC	22	50
**ASO_31B**	CUUCUUGUGGAGUAGAGAUGUU	22	41
**ASO_39A**	AGCCUGGGAGGCAGCAC	17	70
**ASO_39B**	AAUCCUUCUGCAUCGUUAAGCUA	23	58
**ASO_39C**	GAAAUCCAUGGGUGGAGUCG	20	55
**ASO_39D**	CUGGAGUUGGUAUCUGGGA	19	52
**ASO_39E**	AACCUGAAGACUGGUUGGA	19	47
**ASO_39F**	ACUCUAGAAAUCCAUGGG	18	44
**ASO_40A**	UAGCUUAACGAUGCAGAAGGAUU	23	39
**ASO_40B**	GCGGUCAUGAAUGGAAUCCA	20	52
**ASO_40C**	CUUACUGUCCUCUGCGG	17	59
**mmASO_30A**	CACUUUGUAUAACUGUUAAGG	21	33
**mmASO_30E**	GCUAUAUCCAUUUAAAAUACG	19	47
**mmASO_31B**	CUUAUUGUGUAUUAAAGAUGUU	22	22

**mmASO_39D**	CUGAAUUUGAUAUCUGAGA	19	52
**mmASO_40A**	UAGAUUAAUGAUGCAUAAGUAUU	23	21

ASO: antisense oligonucleotide; mm: mismatch; nt: nucleotide; Mismatches with the target sequence are underlined. All ASO were ordered with 2’-O-(2-methoxyethyl) modified ribose groups and a fully phosphorothioated backbone.

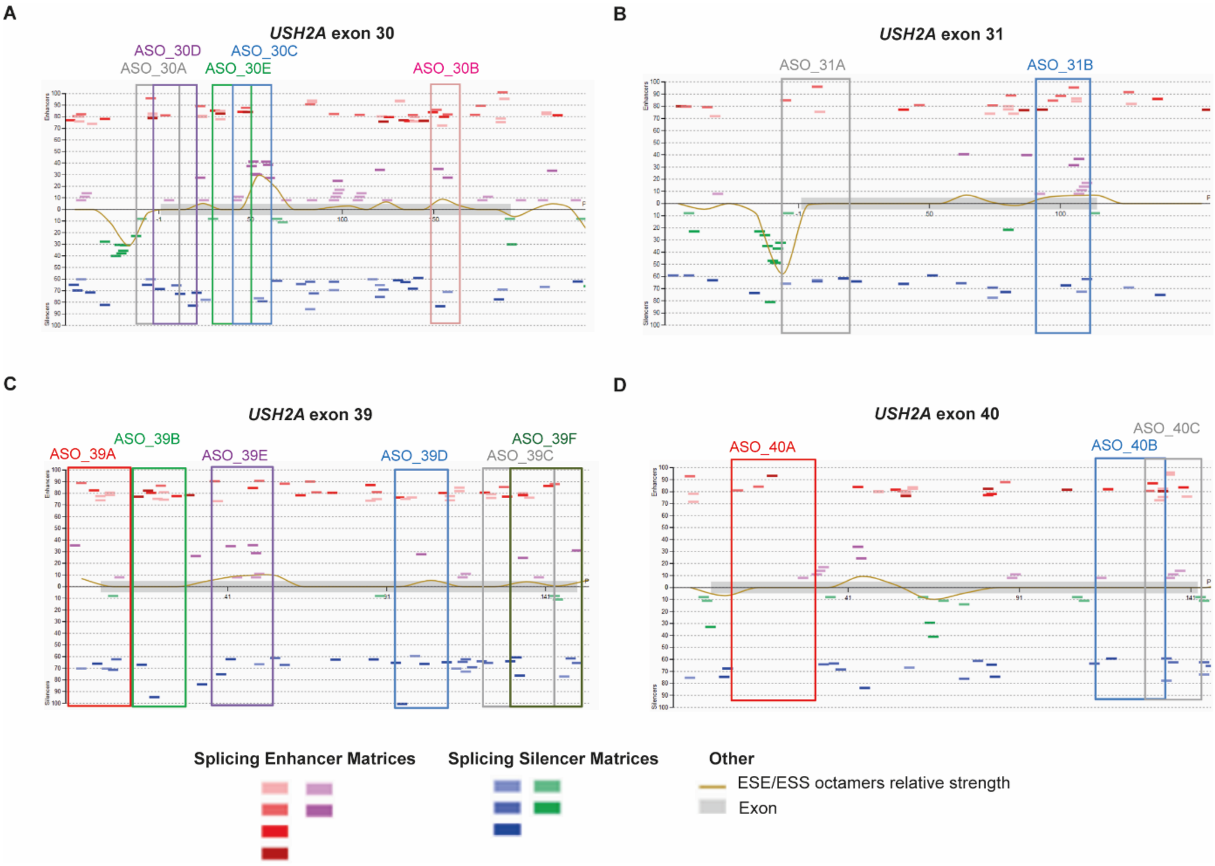

Figure S1. Overview of target sites for the designed antisense oligonucleotide (ASO). ASOs that specifically induce skipping of **(A)** *USH2A* exon 30, **(B)** exon 31, **(C)** exon 39 or **(D)** exon 40 were designed. ASOs target either the intron-exon boundaries, or known exonic splicing enhancer (ESE) motifs within the exons. Splicing Enhancer Matrices and Splicing Silencer Matrices were assessed and visualized using the 'Human Splicing Finder' website (http://www.umd.be/HSF3/).

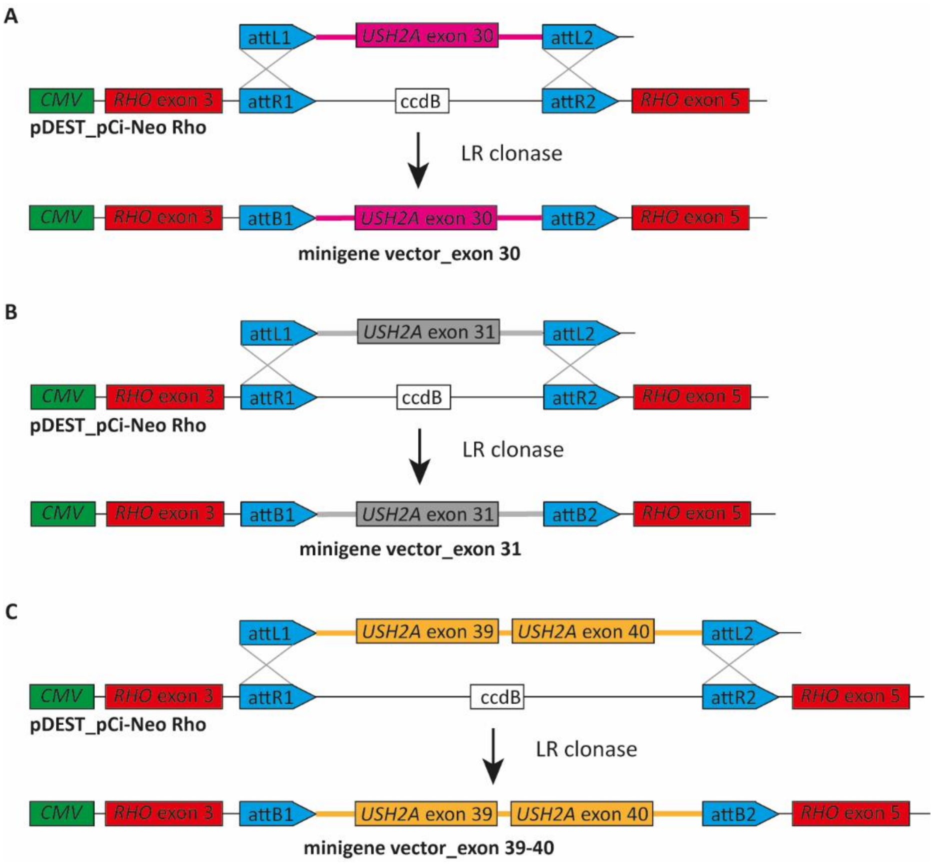

Figure S2. Generation of the minigene splice vectors. The genomic region containing **(A)** *USH2A* exon 30, **(B)** exon 31 or **(C)** exons 39-40 and flanking sequences were cloned into the pCI-Neo Rho destination vector. This resulted in three minigene splice vectors which contain the fragments of interest flanked by two rhodopsin exons under the control of a CMV promotor. pDEST_pCI-Neo Rho: pCi-Neo Rho destination vector.

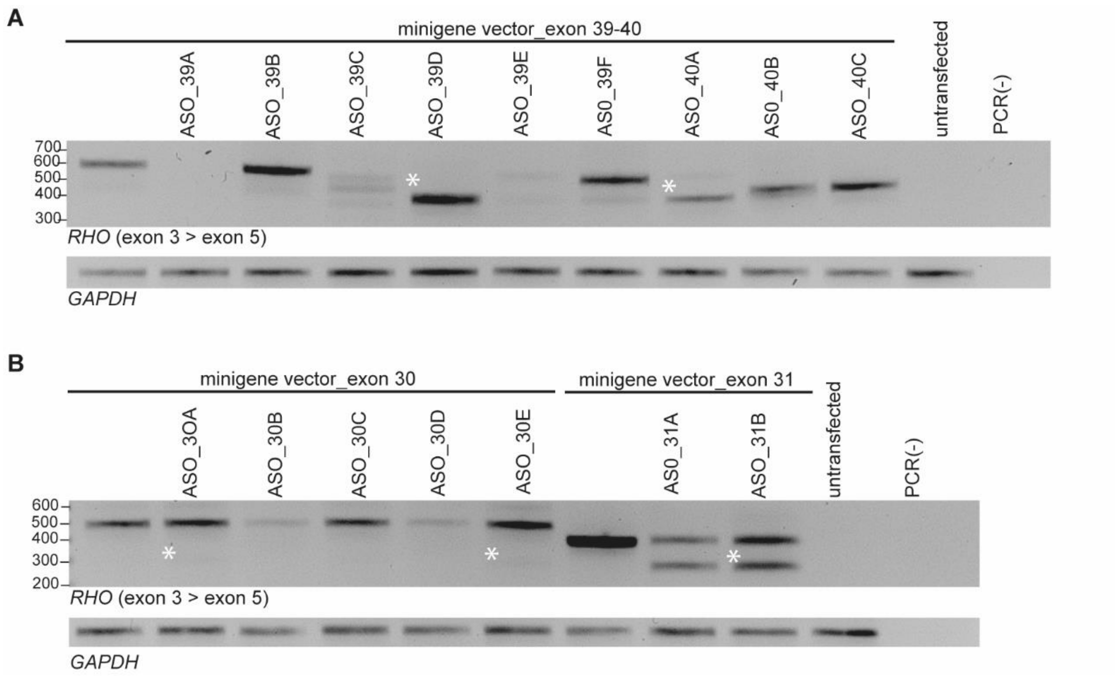

Figure S3. Identification of potent ASOs using a minigene splice assay. HEK293T cells were co-transfected with the minigene splice vector of interest and ASOs targeting *USH2A* exon 30, 31, 39 or 40. Sanger sequencing of amplicons indicated by an asterisk confirmed correct exon skipping. *GAPDH* amplification is shown as a loading control. ASO: antisense oligonucleotide; PCR(-): negative PCR control.

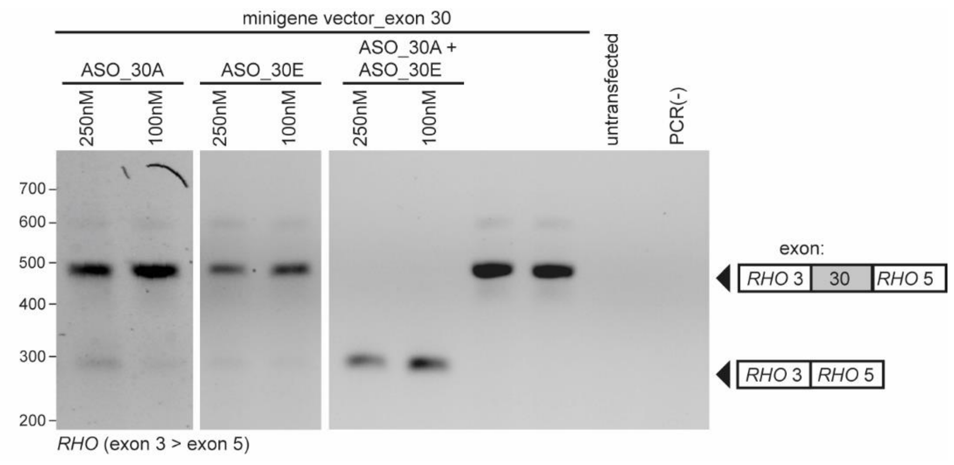

Figure S4. Validation of dual ASO-induced skipping of *USH2A* exon 30 using a minigene splice assay. HEK293T cells were co-transfected with the minigene splice vector and either ASO_30A, ASO_30E or a cocktail of ASO_30A and ASO_30E in a final concentration of 100 or 250 nM. The upper amplicon represents the transcript containing exon 30, whereas the lower amplicon represents the transcript lacking exon 30. ASO: antisense oligonucleotide; PCR(-): negative PCR control.
